# Vitamin D status and tic disorder: a systematic review and meta-analysis of observational studies

**DOI:** 10.3389/fped.2023.1173741

**Published:** 2023-05-30

**Authors:** Lin Xiaoxia, Jiang Jilong, Chen Xianrui, Chen Yanhui

**Affiliations:** ^1^ Department of Pediatrics, Fujian Medical University Union Hospital, Fuzhou, China; ^2^ Department of Pediatric Rehabilitation, Xiamen Rehabilitation Hospital, Xiamen, China

**Keywords:** vitamin D, tic disorders, children, systematic review, meta-analysis

## Abstract

**Objective:**

Tic disorders (TD) are a common neurodevelopmental disorder, it can be divided into transient tic disorder (TTD), chronic motor or vocal tic disorder (CTD), and Tourette syndrome (TS). Our research is to evaluate the clinical relationship between tic disorders and vitamin D level in children.

**Methods:**

Online databases, including CNKI, Wanfang, VIP, Cochrane Library, PubMed and Embase digital knowledge service platform, were checked up to June 2022 for relevant observational studies published in Chinese and English. A random-effects model was incorporated to summarize the study results. The RevMan5.3 software was used for meta-analysis.

**Results:**

Out of 132 retrieved articles, 13 observational studies were eligible for inclusion in the systematic review and meta-analysis, comparing serum Vitamin D levels between children with TD and HC (healthy controls), including different subtypes of TD (TTD, CTD and TS). The results showed that the serum vitamin D levels in the TD group were lower than those in the HC group (MD = −6.64, 95% CI: −9.36 to −3.93, *P* < 0.001, Heterogeneity test: *P* < 0.001, *I*^2^ = 94%). There were no statistically significant differences in serum vitamin D levels between the TTD group and the CTD group (MD = 3.84, 95% CI: −0.59 to 8.26, *P *= 0.09, Heterogeneity test: *P* < 0.001, *I*^2^ = 90%), or between the CTD group and the TS group (MD = 1.06, 95% CI: −0.04 to 2.16, *P* = 0.0, Heterogeneity test: *P* = 0.54, *I*^2^* *= 0%). However, there was a statistically significant difference in serum vitamin D levels between the TTD group and the TS group (MD = 5.24, 95% CI: 0.68–9.80, *P* = 0.02, Heterogeneity test: *P* < 0.001, *I*^2^ = 92%). The study also found a statistically significant difference in the ratio of male children between the TD group and the HC group (OR = 1.48, 95% CI: 1.07–2.03, *P* = 0.02, Heterogeneity test: *P* < 0.001, *I*^2^ = 74%), but no statistically significant difference in the age of children between the TD group and the HC group (OR = 0.46, 95% CI: −0.33 to 1.24, *P* = 0.25, Heterogeneity test: *P* < 0.001, *I*^2^* *= 96%).

**Conclusions:**

Our meta-analysis showed that the vitamin D level of children with TD was lower than that of healthy children. However, there was no difference between the subgroup. Due to the limitations of included studies in research design and diagnostic criteria, large samples, multi-center and high-quality studies are still needed for further analysis and confirmation.

## 1. Introduction

Tic disorders (TD) are a common neurodevelopmental disorder, particularly among children. According to its clinical characteristics and course, it can be divided into transient tic disorder (TTD), chronic motor or vocal tic disorder (CTD), and Tourette syndrome (TS) (1). The prevalence of TD in children and adolescents is approximately 3%, with the age of onset ranging from 4 to 18 years (2, 3). The etiology and pathogenesis of tic disorders are not fully understood. However, several biological, psychological, genetic and environmental risk factors have been proposed (4).

Müller-Vahl et al. (5) have suggested that spontaneous fluctuations in tics may be caused by short-term changes in the dopaminergic system, which plays an important role in the pathogenesis of TD. While vitamin D (VitD) plays a vital role in the normal development and function of the dopaminergic system, it may protect the integrity of dopaminergic neurons by upregulating the secretion of neurotrophic factors and inducing antioxidant effects, and it is hypothesized that TD may be associated with VitD (6–8). It is known that VitD is a class of neuroactive steroids in addition to maintaining the body's calcium and phosphorus metabolic balance (9). Studies have shown that blood levels of vitamin D are positively correlated with nervous system functions such as memory, logical analysis, mood, and balance (10). Pertile et al. (11) have found that VitD directly regulates the expression of tyrosine hydroxylase, a rate-limiting enzyme necessary for the production of dopamine, epinephrine and norepinephrine, which occurs early in rat brain development; prenatal VitD deficiency alters the development of dopaminergic pathways; VitD deficiency predisposes to significant changes in the ratio of DA and 5-HT levels, a decrease in Glu and glutamine (Gln), VD deficiency is likely to lead to significant changes in the ratio of DA and 5-HT, a decrease in Glu and Gln levels and an increase in GABA levels (12). VitD is also a potential modulator of immune activity, which plays a neuroprotective role (13). Although the exact mechanism by which VitD deficiency increases the incidence of TD is not clear, VitD deficiency may indirectly have an adverse effect on overall brain development and function during early embryonic development due to the widespread distribution of VitD receptors, whether in terms of early central nervous system establishment, neurotransmitter synthesis, or neurotrophic factor regulation, thereby increasing the risk of TD.

Several observational studies have examined the association between VitD status and TD in children in recent years; however, they have achieved inconsistent results (14–18). In 2017, Li et al. (19) demonstrated a high prevalence of vitamin D insufficiency or deficiency in children with tic disorders, and there was a negative correlation between the serum 25(OH)D level and tic severity. Bond et al. (17) fond that lower vitamin D levels were not associated with a higher presence or severity of tic disorders but associated with the presence and severity of comorbid ADHD. The study done by Cui et al. (18) has reported that there is no difference in blood VitD between children with TD and healthy controls (HC), and also no differences among subtypes. Notwithstanding that several original studies and some reviews have been undertaken, there is still no consensus on the subject of “a correlation between serum values of VitD and TD”. To achieve these objectives, we conducted a systematic literature review and qualitative evidence synthesis of observational studies, to provide overall estimates and find the potential sources of heterogeneity between the results.

## 2. Materials and methods

The present systematic review and meta-analysis was performed according to the Preferred Reporting Items for Systematic Reviews and Meta-Analyses (PRISMA 2020) guidelines (20). Our meta-analysis was based on published studies, so an ethical statement was not required.

### 2.1. Search strategy

Published studies conducted were searched thoroughly using electronic databases including Wanfang, China National Knowledge Infrastructure (CNKI), Weipu databases, Medline, PubMed, Embase and the Cochrane Library from inception date until June 2022. Besides, the available references of included studies articles and relevant reviews were also reviewed to identify gray literature. The literature searches were performed using Medical Subject Headings (MeSH terms) and also free-text words that might be used in the title and/or abstract of the relevant papers: “TD”, “tic disorders”, “Tourette Syndrome”; “vitamin D”, “calcitriol”, “calcifediol”, “cholecalciferol”, “ergocalciferol”, “25-hydroxyvitamin D”, “25(OH)D”, “1,25(OH)(2)D”, “25-hydroxyvitamin D 2”, “1,25(OH)D”, “25 hydroxyvitamin D”, “25-(OH)D(3)” and “child”. We used the search terms separately and Boolean operators like “OR” or “AND” in combination. The reference lists of the relevant articles were checked for any additional investigation. Publications found were compared and reviewed for their relevance with the use of the pre-specified inclusion and exclusion criteria by two independent authors (XX L and XR C) and disagreements were resolved by group discussion. If any disagreement still remained, it was resolved by discussion with the third author YH C).

### 2.2. Inclusion criteria

Study population: Children were aged ≤14 years.

Study period: The year of data collection of the study was limited by the period from inception date until June 2022.

Study type: Data from all study designs (i.e., Case-control studies, cohort studies, nested case-control studies). Published articles in English and Chinese language were included.

Study outcome indicators: Children met the diagnostic criteria IV for Tic disorder. The study objective was the clinical relationship between VitD and TD. Researches including directly and/or indirectly providing clinical data or indicator were included.

### 2.3. Exclusion criteria

Studies which did not have a clear diagnostic criterion for TD or were conducted in children with other neurological disorders were excluded. Duplicate articles, reviews, case reports, letters, and commentaries were also excluded.

### 2.4. Data extraction and quality assessment

Two sorts of reference management software (NoteExpress V3.0 and Endnote version X7.0) were used to remove duplicate articles. Two authors (XX L and XR C) used a standardized data extraction format on WPS excel spreadsheet to extract the data independently, and the accuracy of the data entry was double-checked and confirmed by the third author (JL J). The data extraction checklist included the key outcome (number of cases, age, genders and blood vitamin D level in the case and control groups), author name, date of publication, screening year, study design, study sample size, and the use of any matching or adjustment for confounding variables in the data analysis.

We used the Newcastle-Ottawa Scale (NOS) to assess the quality of studies included in the systematic review (21). The scale considers 3 major domains: the selection of the study groups (4 items); the comparability of the study groups (1 item); and the ascertainment of either the exposure or the outcome (3 items) for the case-control and cohort studies, respectively. A study receives a maximum of 1 star (score) for each item of the selection and outcome or exposure domains. However, it receives a maximum of 2 stars for the item designed to assess comparability. Therefore, a study might receive a total score of 9 using this tool. 9 assessment criteria were included with three potential responses: “Yes”, “No” or “Unclear”. In the present study, studies scoring ≥5 were considered to be high quality, and those with scores ≤4 were considered as low quality, respectively. The adopted Agency of Healthcare Research and Quality (AHRQ) assessment tool was used to assess each included original study by two authors (XR C and XX L) independently. During the time of data extraction and Quality assessment, discrepancies between two independent authors were resolved through a third author (YH C) after discussion and consensus.

### 2.5. Statistical analysis

The meta-analysis was performed with the Review Manager 5.3 software and STATA, version 11.2 (Stata Corp.), the sample sizes and the mean ± SD for serum 25(OH)D concentrations in participants with and without TD were obtained to derive the mean difference ± SD in serum VitD concentrations between TD groups and HC groups. This was then used as the effect size for the meta-analysis of the means. Furthermore, count data were represented by the risk ratio (odds ratio, OR), with each effect size being a point estimated with its 95% confidence interval (confidence intervals, CI). The Cochran chi-square-based *Q* test and the *I*^2^ Test were used to examine the heterogeneity of the studies (19). If there was no statistical heterogeneity (*I*^2^ ≤ 50%), a fixed-effects model was used for meta-analysis, otherwise, a random-effects model was used for analysis. Subgroup analysis and sensitivity analysis were used to explore the heterogeneity. Publication bias was assessed by visually checking funnel plots and conducting Egger's regression and Begg's adjusted rank correlation asymmetry tests (22–24). *P* value < 0.05 was considered significant.

## 3. Results

### 3.1. Study selection and characteristics of the included studies

**Figure 1:**
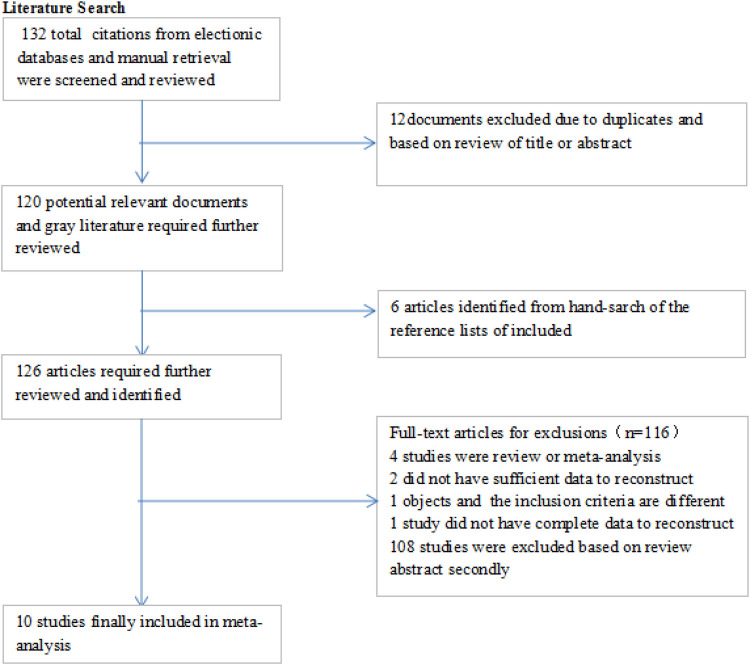
The flow chart of the selection process.

The database search and manual searches led to 132 and 6 publications, respectively. After removing duplicates, 126 articles were screened for the titles and abstracts, which resulted the exclusion of 116 publications. In total, 10 relevant studies (15–19, 25–29) remained to be included in the current systematic review and meta-analysis (17). The flow chart of the selection process is shown in Figure 1. The characteristics of the included studies in the systematic review and meta-analysis are shown in Table 1.

**Table 1:** The characteristics of the included studies.

Author	Country	Ntotal (TD/HC)	Nmale (TD/HC)	Age (year)		Serum 25(OH)D levels (ng/ml)		NOS
				TD	HC	TD	HC	
Chen 2019 (25)	China	111/111	64/63	6.82 ± 2.28	7.35 ± 2.19	22.60 ± 2.86	33.28 ± 2.42	7
Hou 2020 (26)	China	245/63	194/50	7.81 ± 2. 67	7.36 ± 2.41	23.72 ± 8.87	26. 61 ± 7. 59	7
Li 2018 (19)	China	179/189	148/154	7.98 ± 0.65	8.00 ± 0.74	22.9 ± 7.5	28.9 ± 8.3	7
Cui 2017 (18)	China	85/60	64/28	7.02 ± 2.86	6.55 ± 1.75	21.34 ± 9.44	22.09 ± 10.16	6
Bian 2020 (27)	China	50/50	31/29	8.41 ± 1.39	8.22 ± 1.51	20.3 ± 5.47	27.91 ± 6.85	5
Ge 2020 (28)	China	130/121	97/89	8.10 ± 2.20	8.50 ± 2.40	22.82 ± 7.17	35.27 ± 7.62	6
Bond 2022 (17)	England	327/93	247/37	10.9 ± 2.72	7.68 ± 1.76	24.7 (19.2–29.8)	21.6 (17.0–27.3)	6
Wang 2022 (15)	China	2,960/2,665	2,355/1,912	NA	NA	38.47 (23.56–58.89)	50.05 (34.47–65.80)	6
Wang 2022 (16)	China	59/55	36/35	7.30 ± 2.08	7.25 ± 2.11	23.25 ± 8.20	26.69 ± 7.52	6
Jia 2022 (29)	China	54/54	44/42	6.33 ± 2.16	5.69 ± 2.20	19.42 ± 9.10	27.49 ± 8.30	6

HC, healthy control; NOS, Newcastle-Ottawa Scale; N, number; NA, not available; TD, tic disorders.

### 3.2. Quality assessment

Both case and control groups in each study were clearly defined, with good representation of cases. All studies had methods to clarify exposure factors, and comparability between case and control groups was considered in the design and statistics, and the most important confounding factors were well controlled. All studies used the same method to measure exposure factors between groups while specifying exposure factors, but there was some selection bias in the control group. All studies had reliable outcome measures, but no non-response rates were described. All studies completed follow-up and had a low rate of missed visits. The quality score of studies ranged from 5 to 7, based on the NOS. Overall, 1 study was assessed to be low quality (27), and the other studies were high quality (15–19, 25, 26, 28, 29) (Table 1).

### 3.3. Meta-analysis of mean serum vitamin D concentrations

#### 3.3.1. TD and HC

**Figure 2:**
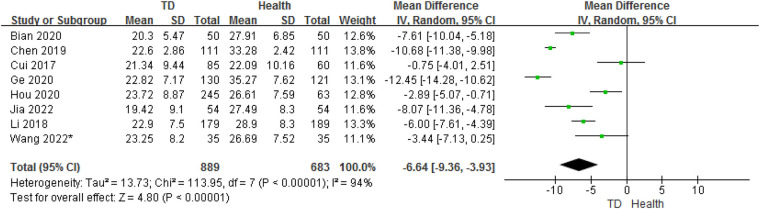
Forest plot of serum vitamin D between the TD and HC.

In total, the meta-analysis of 8 studies showed that children with TD had 6.64 ng/ml lower serum vitamin D concentrations compared with HC (95% CI: −9.36 to −3.93; *P* < 0.001) with high heterogeneity (*P* < 0.001, *I*^2^ = 94%) (Figure 2). Sensitivity analysis revealed that removing each study included in the meta-analysis did not substantially change the overall estimate.

#### 3.3.2. TTD and CTD groups

**Figure 3:**
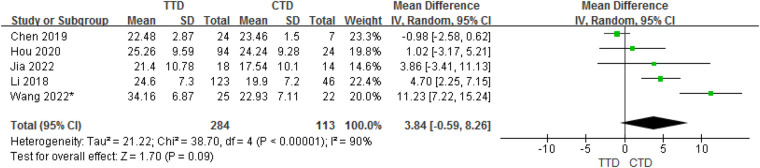
Forest plot of serum vitamin D between the TTD and CTD groups.

In total, 5 studies provided data on the mean ± SD vitamin D concentrations in subjects with TTD and CTD. Although the meta-analysis of their results showed that children with TTD had 3.84 ng/ml higher serum vitamin D concentrations compared with CTD, but in fact, there is no statistical difference between them (95% CI:−0.59 to 8.26, *P* = 0.09). The heterogeneity between studies was significant (Cochran's *Q* test, *P* < 0.001; *I*^2^ = 90%) (Figure 3).

#### 3.3.3. CTD and TS groups

**Figure 4:**
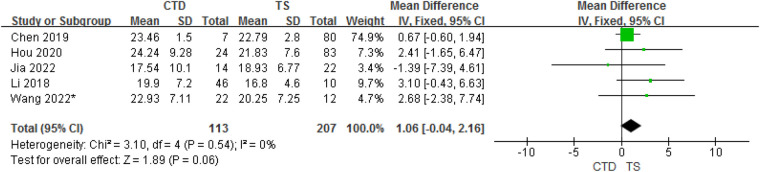
Forest plot of serum vitamin D between the CTD and TS groups.

In total, 5 studies provided data on the mean ± SD vitamin D concentrations in subjects with CTD and TS. Although the meta-analysis of their results showed that children with CTD had 1.06 ng/ml higher serum vitamin D concentrations compared with TS, but in fact, there is no statistical difference between them (95% CI: −0.04 to 2.16, *P* = 0.06). The heterogeneity between studies was not significant (Cochran's *Q* test: *P* = 0.54; *I*^2^ = 0%) (Figure 4).

#### 3.3.4. TTD and TS groups

**Figure 5:**
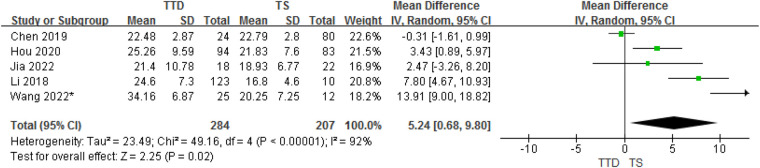
Forest plot of serum vitamin D between the TTD and TS groups.

In total, the meta-analysis of 8 studies showed that children with TTD had 5.24 ng/ml higher serum vitamin D concentrations compared with TS (95% CI: 0.68–9.80; *P* = 0.02) with high heterogeneity (*P* < 0.001, *I*^2^ = 92%). The heterogeneity between studies was significant (Cochran's *Q* test: *P* < 0.001; *I*^2^ = 92%) (Figure 5).

### 3.4. Analysis of sex between TD and HC

**Figure 6:**
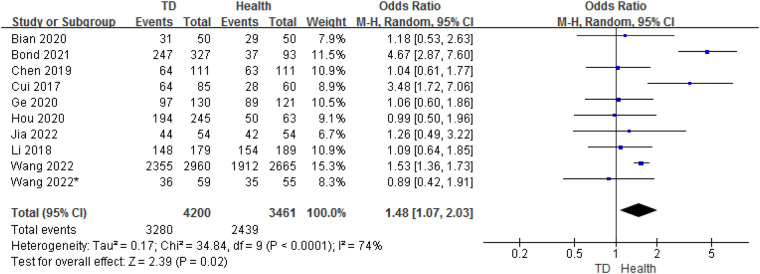
Comparison of sex between the TD group and the control group.

In total, 10 studies provided gender data on the ORs for TD in children compared with HC. The analysis of ORs showed a significant association between male and the likelihood of TD (OR = 1.48, 95% CI: 1.07–2.03, *P* = 0.02) (Figure 6). The heterogeneity between studies was significant (Cochran's *Q* test: *P* < 0.001; *I*^2^ = 74%).

### 3.5. Analysis of age between TD and HC

**Figure 7:**
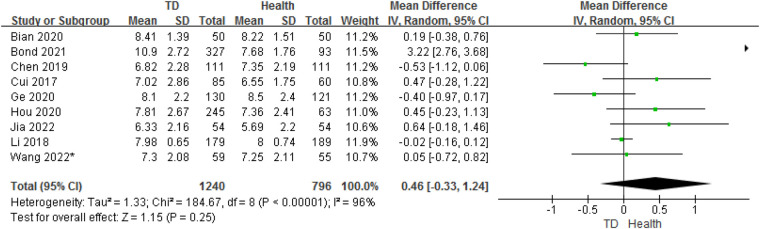
Comparison of age between the TD group and the control group.

In total, 9 studies provided age data on the ORs for TD in children compared with HC. The analysis of ORs showed nosignificant association between TD and HC (OR = 0.46, 95% CI: −0.33 to 1.24, *P* = 0.25) (Figure 7). The heterogeneity between studies was significant (Cochran's *Q* test: *P* < 0.001; *I*^2^ = 96%).

### 3.6. Pooled prevalence of vitD deficiency

**Figure 8:**
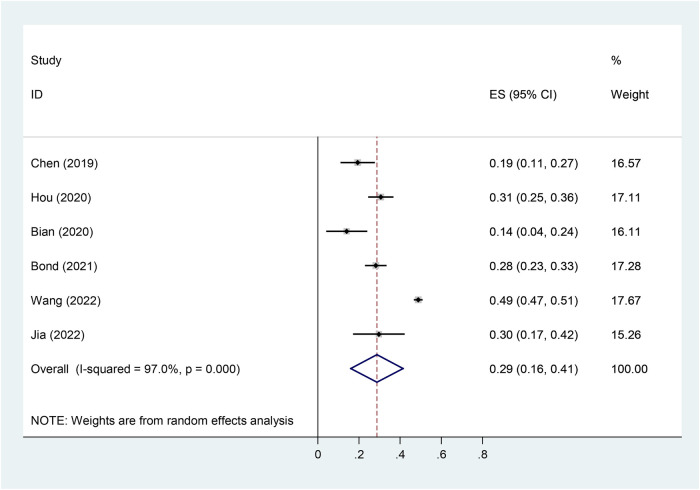
Forest plot of vitD deficiency in TD.

In our meta-analysis, six studies reported the incidence of vitamin D deficiency [serum 25 (OH) D <20 ng/ml] in children with Tourette's syndrome, with a heterogeneity test *P* < 0.001, *I*^2^ = 97%. Using a random-effects model analysis, the meta-analysis results showed that the overall deficiency rate of vitamin D was 28.7% (95% CI = 16.0–41.5%) (Figure 8).

### 3.7. Publication bias

**Figure 9:**
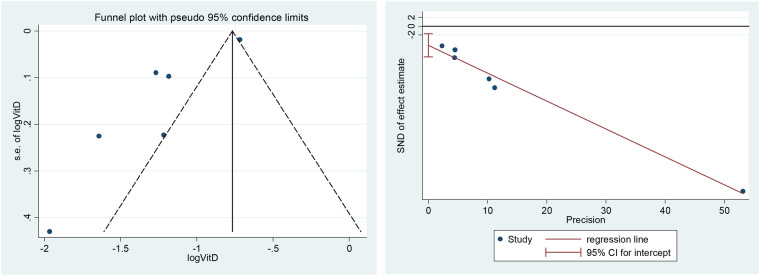
Funnel plot (left) and Egger's tests for publication bias (right).

A funnel plot including all studies did show evidence of asymmetry. The analysis results of Egger's tests were also consistent (*t* = −4.59, *P* = 0. 01). However, we still identified publication bias in some subgroups (Figure 9).

## 4. Discussion

Our meta-analysis identifies that the concentrations of serum 25(OH)D in children with TD are significantly reduced compared to the healthy individuals. The observed results appear to be independent of subtypes of TD, as well as genders and ages. VitD supplementation has been proven to be beneficial for the therapy of CTD (19) (Figure 9).

The reasons for the reduction of VitD and its role in TD pathogenesis remain unclear. VitD is primarily produced in the skin when exposed to ultraviolet light and can be obtained naturally from small amounts of food (30, 31). The synthesis of VitD is greatly affected by some factors, such as season, air pollution, latitude, as well as genetic factors, and altered absorption/metabolism (31). Our study showed that the level of serum vitamin D in children with TD is lower than that of the healthy control group. And the analysis of ORs showed a significant association between male and the likelihood of TD. However, these result have high heterogeneity. In order to strictly control for the influencing factors of vitamin D, Li et al. (32) matched TD patients and control group children in terms of age, visiting season, and other factors. The study results showed that despite both groups of children being affected by the above factors, the serum 25(OH)D levels of TD patients were still significantly lower than those of the control group. Different studies included in this meta-analysis matched for age, visiting season, and other factors, but the vitamin D nutritional status of children in different regions may be closely related to sunlight exposure time/intensity, individual nutritional status, dietary habits, and economic and cultural factors, which may also be one of the possible sources of heterogeneity in the results of this meta-analysis. Roughly 40% of the world's population is deficient in vitamin D, with 25(OH)D levels below 50 nmol/L. Additionally, 60% of the world population has insufficient vitamin D levels ranging from 50 to 79 nmol/L (33). The European Society of Gastroenterology, Hepatology, and Nutrition recommends that 25(OH)D levels below 25 nmol/L are considered deficient, while levels above 50 nmol/L are considered sufficient (34). The United States Endocrine Society recommends substantially higher levels of above 75 nmol/L, based on a suggested plateau in parahormone status and an observed optimum for non-vertebral fracture prevention and intestinal calcium absorption (35). In different regions of China, there are significant differences in vitamin D nutritional status. In the northern region, the serum 25-(OH)D levels of children are between 40 and 50.25 nmol/L (16.0–20.1 ng/ml), and the deficiency rate of vitamin D in children is around 30%–70%. In the southern region, the serum 25-(OH)D levels of children are between 52 and 124 nmol/L (20.8–49.6 ng/ml), and the deficiency rate of vitamin D in children is around 10%–40% (36). Children with TD experienced more challenges in eating behaviour (37, 38). Some drugs for TD could affect the synthesis of VitD (14, 39). These might impact vitamin D status in the blood. But there was no more study conducted on other factors in TD. Future research should focus more on the reasons for the reduction of VitD in TD.

As a neurosteroid hormone, VitD could modulate cell differentiation, axon growth, and the production of neurotransmitters, reactive oxygen species and nerve growth factor in the brain through numerous pathways (31). VitD was able to regulate several transmitter systems that were involved in the pathogenesis of TD. The level of gamma-aminobutyric acid (GABA) has been reported be elevated in rat brains with a chronic VitD deficiency. More research, however, has previously focused on the dopaminergic system. VitD receptors are widely expressed in the brain, mainly distributed in dopamine-rich regions (40, 41). Although dopamine level is shown to be normal in the forebrain of VitD deficient rats, the dopaminergic metabolic profile is abnormal, with an increased ratio of dihydroxyphenylacetic acid (DOPAC)/homovanillic acid (HVA) (42). VitD deficient rats have showed high sensitivity to amphetamine-induced locomotion, with elevated dopamine transporter density (43). Haloperidol, an effective dopaminergic agent for TD, has been identified to improve unnormal behaviours in VitD deficient rats (44).

The inflammatory response, one of the pathogeneses of TD (1), is a vital mechanism of VitD in diseases (45). VitD, with its immunomodulatory properties, modulates not only peripheral inflammation, but also central nervous system (CNS) inflammation (45). VitD receptors are also expressed in microglia (46). According to some researches, maintaining circulating concentrations of 25(OH) D within the range of 100–150 nmol/L appears to optimize the effectiveness of vitamin D in improving immune function. This can substantially reduce the risk of serious infections, particularly from severe acute respiratory syndrome coronavirus 2 (SARS-CoV-2). The hypothesized mechanism for this involves modulation of the immune response, which can help prevent a dangerous and often fatal cytokine storm (33) VitD restriction in mice has been shown to induce morphological changes, with increasing activation of microglia (47). VitD can inhibit the expressions of nuclear factor κB and its downstream inflammatory factors, interleukin-1β (IL-1β), IL-6 and tumour necrosis factor-α (TNF-α), in microglia (46, 48). Some evidence support that microglia are activated in the CNS of subjects with TD, accompanied by the up-regulation of TNF-α and IL-6 expressions (49, 50). It suggests that VitD may mediate the pathogenesis of TD by modulating the levels of inflammatory factors. However, whether vitamin D supplementation affects the levels of inflammatory factors in TD patients remains of concern and requires further study.

In addition, it was noticed that there was high heterogeneity in the results. We did not find statistical differences in age between the TS and HC groups, and the level of serum Vitamin D concentrations between CTD and TTD group, and between CTD and TTD. But children with TTD had higher serum vitamin D concentrations compared with TS. Only one study involved the English population, making it impossible to compare by ethnicity. Although high-performance liquid chromatography, the preferred method for measuring VitD, was used in most of the included studies, VitD binding protein levels should still be considered a potential factor for interfering with VitD status (51). Considering that different drugs have varying effects on VitD levels, there was no medication information in the included studies. Due to research limitations, patients were selected based on the diagnostic and exclusion criteria for TD, and there was no systematic investigation of TD complications, particularly those related to mental and psychological disorders. Therefore, our study lacks relevant data on the correlation between vitamin D deficiency and TD complications. Additionally, we hope that future studies on vitamin D supplementation will address the lack of longitudinal data on treatment follow-up for TD in this project. Despite employing a random-effects model, subgroup analysis, and meta-regression analysis, we were unable to identify the factors responsible for the observed heterogeneity. Additionally, our study only included case-control studies, which are considered less reliable than cohort studies. Therefore, we urge caution in interpreting our findings based on these limitations.

## 5. Conclusions

Our study indicates that there is an association between VitD status and TD. The significance of VitD deficiency in TD still needs to be treated with caution due to the high heterogeneity among studies and an uncertain causality. Considering the high incidence of vitamin D deficiency in subjects with TD, screening and proper interventions for VitD may be significant for TD. It is essential to clarify the causal relationship between the low status of VitD and TD and the underlying mechanisms by further investigations.

## Data Availability

The original contributions presented in the study are included in the article, further inquiries can be directed to the corresponding authors.
